# Prop-2-en-1-yl 4-(4,5-diphenyl-1*H*-imidazol-2-yl)benzoate

**DOI:** 10.1107/S160053681301619X

**Published:** 2013-06-15

**Authors:** Shaaban K. Mohamed, Mehmet Akkurt, Adel A. Marzouk, Antar A. Abdelhamid, Francisco Santoyo-Gonzalez

**Affiliations:** a Chemistry and Environmental Division, Manchester Metropolitan University, Manchester M1 5GD, England; b Chemistry Department, Faculty of Science, Mini University, 61519 El-Minia, Egypt; c Department of Physics, Faculty of Sciences, Erciyes University, 38039 Kayseri, Turkey; d Pharmaceutical Chemistry Department, Faculty of Pharmacy, Al Azhar University, Egypt; e Chemistry Department, Faculty of Science, Sohag University, 82524 Sohag, Egypt; f Department of Organic Chemistry, Faculty of Science, Institute of Biotechnology, Granada University, Granada, E-18071, Spain

## Abstract

The title compound, C_25_H_20_N_2_O_2_, crystallized with two mol­ecules in the asymmetric unit, in one of which the atoms of the terminal propenyl group are disordered over two sets of sites, with a refined occupancy ratio of 0.870 (4):0.130 (4). The central imidazole ring makes dihedral angles of 25.51 (11), 40.73 (11) and 27.36 (11)° with the three pendant rings in one molecule and 22.56 (10), 60.72 (10) and 5.85 (10)° in the other. In the crystal, mol­ecules are linked by N—H⋯N and C—H⋯O hydrogen bonds, forming a three-dimensional network. The crystal structure also features C—H⋯π inter­actions and π–π stacking [centroid–centroid distances = 3.8834 (18) and 3.9621 (17) Å] inter­actions.

## Related literature

For the synthesis and biological activity of imidazole compounds, see, for example: Bhatnagar *et al.* (2011 ▶); Sisko & Mellinger (2002 ▶). For similar structures, see: Akkurt *et al.* (2013*a* ▶,*b* ▶); Mohamed *et al.* (2013*a* ▶,*b* ▶).
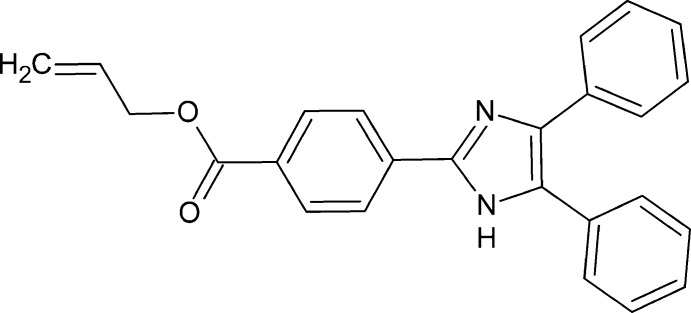


## Experimental

### 

#### Crystal data

C_25_H_20_N_2_O_2_
*M* *_r_* = 380.43
Monoclinic, 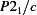

*a* = 15.705 (5) Å
*b* = 14.888 (5) Å
*c* = 17.589 (6) Å
β = 105.151 (4)°
*V* = 3970 (2) Å^3^
*Z* = 8
Mo *K*α radiation
μ = 0.08 mm^−1^
*T* = 100 K
0.73 × 0.35 × 0.15 mm

#### Data collection

Bruker SMART APEX CCD area-detector diffractometer
Absorption correction: multi-scan (*SADABS*; Sheldrick, 2004 ▶) *T*_min_ = 0.967, *T*_max_ = 0.988
46196 measured reflections
9345 independent reflections
6507 reflections with *I* > 2σ(*I*)
*R*_int_ = 0.064

#### Refinement

*R*[*F* ^2^ > 2σ(*F* ^2^)] = 0.058
*wR*(*F* ^2^) = 0.149
*S* = 1.02
9345 reflections
530 parameters
42 restraints
H-atom parameters constrained
Δρ_max_ = 0.51 e Å^−3^
Δρ_min_ = −0.47 e Å^−3^

### 

Data collection: *SMART* (Bruker, 2001 ▶); cell refinement: *SAINT* (Bruker, 2001 ▶); data reduction: *SAINT*; program(s) used to solve structure: *SIR97* (Altomare *et al.*, 1999 ▶); program(s) used to refine structure: *SHELXL97* (Sheldrick, 2008 ▶); molecular graphics: *ORTEP-3 for Windows* (Farrugia, 2012 ▶); software used to prepare material for publication: *WinGX* (Farrugia, 2012 ▶) and *PLATON* (Spek, 2009 ▶).

## Supplementary Material

Crystal structure: contains datablock(s) global, I. DOI: 10.1107/S160053681301619X/hg5323sup1.cif

Structure factors: contains datablock(s) I. DOI: 10.1107/S160053681301619X/hg5323Isup2.hkl

Supplementary material file. DOI: 10.1107/S160053681301619X/hg5323Isup3.cml

Additional supplementary materials:  crystallographic information; 3D view; checkCIF report

## Figures and Tables

**Table 1:** Hydrogen-bond geometry (Å, °) *Cg*1, *Cg*2 and *Cg*3 are the centroids of the N1/N2/C1–C3 1*H*-imidazole ring and the C4–C9 and C10–C15 phenyl rings, respectively.

*D*—H⋯*A*	*D*—H	H⋯*A*	*D*⋯*A*	*D*—H⋯*A*
N1—H1*N*⋯N4^i^	0.90 (2)	2.14 (2)	3.037 (2)	176 (2)
N3—H3*N*⋯N2	0.89 (2)	2.06 (2)	2.925 (2)	164 (2)
C7—H7⋯O1^ii^	0.95	2.45	3.254 (3)	142
C32—H32⋯O3^iii^	0.95	2.51	3.439 (3)	167
C23—H23*A*⋯*Cg*1^iv^	0.99	2.74	3.524 (3)	136
C25*A*—H25*B*⋯*Cg*2^v^	0.95	2.79	3.687 (3)	158
C37—H37⋯*Cg*3^vi^	0.95	2.90	3.634 (3)	134
C25*B*—H25*D*⋯*Cg*2^v^	0.95	2.68	3.44 (2)	137

Symmetry codes: (i) 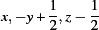; (ii) 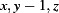; (iii) 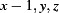; (iv) 
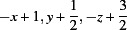
; (v) 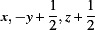; (vi) 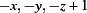.

**Fig. 1.**
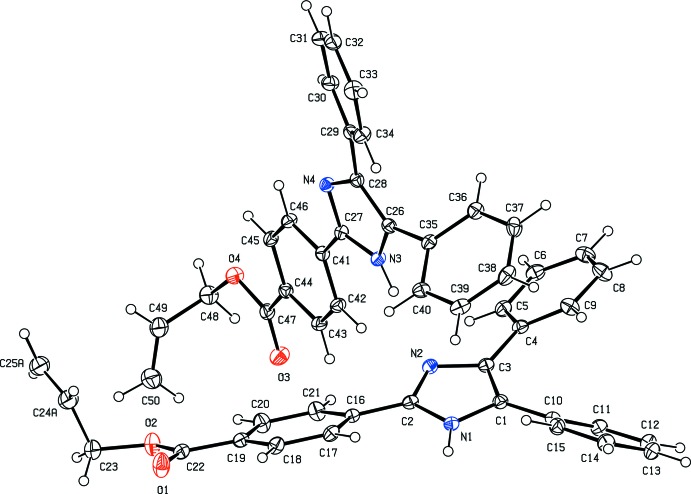
The molecular structure of (I) with the atom numbering scheme. Displacement ellipsoids for non-H atoms are drawn at the 30% probability level. Only the major components of disorder are shown.

**Fig. 2.**
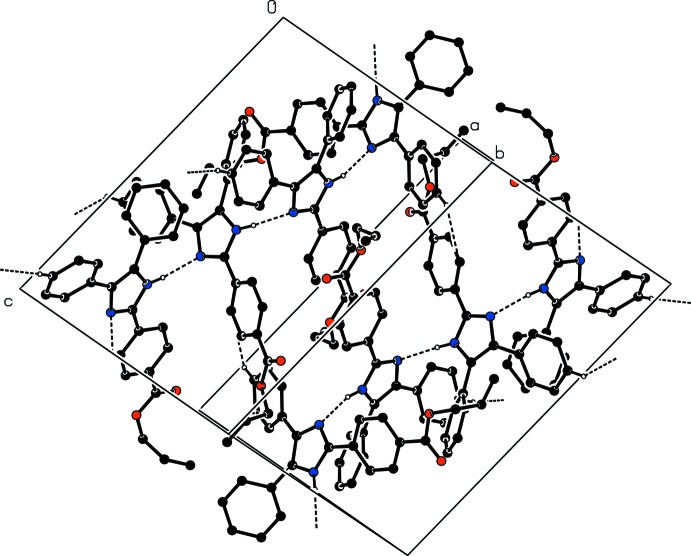
A part of the hydrogen bonding and packing of (I) viewing along the [-110]-axis. H atoms not involved in hydrogen bonds and the minor components of disorder have been omitted for clarity.

## supplementary crystallographic information

### Comment

Imidazole compounds represent a wide range of bio-activities such as anti-fungal, anti-inflammatory, anti-anthelmintic, anti-viral and anti-ulcer activities in addition to their use in treatment of rheumatoid arthritis (Bhatnagar *et al.*, 2011; Sisko & Mellinger, 2002). Based on such facts and following to our ongoing study on synthesis of unsaturated substituted imidazoles as a convenient substrate to design bioactive molecules, we herein report the synthesis and crystal structure of the title compound.

Fig. 1 shows the two molecules A (with N1) and B (with N3) of the title compound (I), in the asymmetric unit. In molecule A, the 1*H*-imidazole ring (N1/N2/C1–C3) makes dihedral angles of 25.51 (11), 40.73 (11) and 27.36 (11)°, respectively, with the two phenyl rings (C4–C9 and C10–C15) and the benzene ring (C16–C21). In molecule B, due to the different molecular environment in the crystal, the corresponding angles are different, *viz.* 22.56 (10), 60.72 (10) and 5.85 (10)°, respectively. In molecule A, the phenyl rings (C4–C9 and C10–C15) form dihedral angles of 21.36 (10) and 26.58 (10) °, respectively with the benzene ring (C16–C21), while the dihedral angle between them is 47.42 (10)°. In molecule B, the corresponding angles are different, *viz.*16.94 (10), 60.11 (9) and 65.48 (10)°. The bond lengths in (I) are normal and are comparable to those reported for similar compounds (Akkurt *et al.*, 2013*a*,*b*; Mohamed *et al.*, 2013*a*,*b*).

Intermolecular N—H···N and C—H···O hydrogen bonds link the adjacent molecules, into a three dimensional network structure (Table 1, Fig. 2). C—H···π interactions (Table 1) and π-π stacking interactions [*Cg*1···*Cg*7(*x*, *y*, *z*) = 3.8834 (18) Å and *Cg*8···*Cg*8(1 - *x*, -*y*, 2 - *z*) = 3.9621 (17) Å; where *Cg*1 is the centroid of the 1*H*-imidazole ring (N1/N2/C1–C3) of molecule A; *Cg*7 and *Cg*8 are the centroids of the phenyl (C35–C40) and benzene (C41–C46) rings of molecule B] are significant contributors in the stabilization of the crystal packing of (I).

### Experimental

A solution of 10 mmol (570 mg) potassium hydroxide in 25 ml dimethyl sulfoxide was charged in a 50-ml volumetric flask equipped with a magnetic stirring bar. The mixture was stirred at room temperature for 5 minutes followed by addition of 10 mmol (3.40 g) 4-(4,5-diphenyl-1*H*-imidazol-2-yl)benzoic acid with stirring for further 30 minutes. A solution of 10 mmol (1.21 g) allyl bromide was added dropwise with stirring for additional 45 minutes. The reaction mixture was diluted with 20 ml water. The mixture was extracted with 3x20 ml diethyl ether. The combined ether layers were dried over calcium chloride. The excess solvent was removed under slightly reduced pressure and the excess allyl bromide was removed by distillation at approximately 15 mm. The raw product was collected and crystallized from ethanol to give the title compound. Single crystals suitable for X-ray diffractions were grown up by slow evaporation of an ethanol solution for the title compound at room temperature.

### Refinement

The H atoms attached to the C atoms were placed in geometrically, with C—H = 0.95–0.99 Å, and refined as riding with *U*_iso_(H) = 1.2*U*_eq_(C). The H atoms of the two amide groups were located from a difference Fourier map and refined with riding model constraints and *U*_iso_(H) = 1.2*U*_eq_(N). The atoms of the terminal propenyl group are disordered over two sites with refined occupancies of 0.870 (4) and 0.130 (4).

### Crystal data

**Table:**

C_25_H_20_N_2_O_2_	*F*(000) = 1600
*M**_r_* = 380.43	*D*_x_ = 1.273 Mg m^−^^3^
Monoclinic, *P*2_1_/*c*	Mo *K*α radiation, λ = 0.71073 Å
Hall symbol: -P 2ybc	Cell parameters from 5599 reflections
*a* = 15.705 (5) Å	θ = 2.4–24.2°
*b* = 14.888 (5) Å	µ = 0.08 mm^−^^1^
*c* = 17.589 (6) Å	*T* = 100 K
β = 105.151 (4)°	Prism, colourless
*V* = 3970 (2) Å^3^	0.73 × 0.35 × 0.15 mm
*Z* = 8	

### Data collection

**Table:**

Bruker SMART APEX CCD area-detector diffractometer	9345 independent reflections
Radiation source: sealed tube	6507 reflections with *I* > 2σ(*I*)
Graphite monochromator	*R*_int_ = 0.064
phi and ω scans	θ_max_ = 28.1°, θ_min_ = 1.3°
Absorption correction: multi-scan (*SADABS*; Sheldrick, 2004)	*h* = −20→19
*T*_min_ = 0.967, *T*_max_ = 0.988	*k* = −19→19
46196 measured reflections	*l* = −23→23

### Refinement

**Table:**

Refinement on *F*^2^	42 restraints
Least-squares matrix: full	Hydrogen site location: mixed
*R*[*F*^2^ > 2σ(*F*^2^)] = 0.058	H-atom parameters constrained
*wR*(*F*^2^) = 0.149	W = 1/[Σ^2^(*F*O^2^) + (0.0551*P*)^2^ + 2.4694*P*] where *P* = (*F*O^2^ + 2*F*C^2^)/3
*S* = 1.02	(Δ/σ)_max_ < 0.001
9345 reflections	Δρ_max_ = 0.51 e Å^−^^3^
530 parameters	Δρ_min_ = −0.47 e Å^−^^3^

### Special details

**Table:**

Geometry. Bond distances, angles *etc*. have been calculated using the rounded fractional coordinates. All su's are estimated from the variances of the (full) variance-covariance matrix. The cell e.s.d.'s are taken into account in the estimation of distances, angles and torsion angles
Refinement. Refinement on *F*^2^ for ALL reflections except those flagged by the user for potential systematic errors. Weighted *R*-factors *wR* and all goodnesses of fit *S* are based on *F*^2^, conventional *R*-factors *R* are based on *F*, with *F* set to zero for negative *F*^2^. The observed criterion of *F*^2^ > σ(*F*^2^) is used only for calculating -*R*-factor-obs *etc*. and is not relevant to the choice of reflections for refinement. *R*-factors based on *F*^2^ are statistically about twice as large as those based on *F*, and *R*-factors based on ALL data will be even larger.

### Fractional atomic coordinates and isotropic or equivalent isotropic displacement parameters (Å^2^)

**Table:**

	*x*	*y*	*z*	*U*_iso_*/*U*_eq_	Occ. (<1)
O1	0.35999 (12)	0.64057 (10)	0.76160 (9)	0.0464 (6)	
O2	0.46517 (10)	0.56399 (10)	0.84766 (9)	0.0386 (5)	
N1	0.26812 (10)	0.22756 (11)	0.54074 (9)	0.0226 (5)	
N2	0.31920 (10)	0.15565 (11)	0.65403 (9)	0.0230 (5)	
C1	0.25545 (12)	0.13717 (13)	0.52362 (11)	0.0229 (6)	
C2	0.30723 (12)	0.23573 (13)	0.61920 (11)	0.0222 (5)	
C3	0.28623 (12)	0.09338 (13)	0.59526 (11)	0.0224 (5)	
C4	0.28431 (12)	−0.00269 (13)	0.61516 (11)	0.0237 (6)	
C5	0.34480 (13)	−0.03739 (14)	0.68160 (11)	0.0269 (6)	
C6	0.34347 (14)	−0.12801 (14)	0.70058 (12)	0.0299 (6)	
C7	0.28312 (14)	−0.18565 (14)	0.65308 (13)	0.0321 (7)	
C8	0.22224 (14)	−0.15158 (15)	0.58780 (13)	0.0349 (7)	
C9	0.22164 (13)	−0.06108 (14)	0.56960 (12)	0.0300 (6)	
C10	0.21672 (13)	0.10645 (13)	0.44218 (11)	0.0247 (6)	
C11	0.24996 (13)	0.03092 (14)	0.41210 (12)	0.0290 (6)	
C12	0.21196 (15)	0.00247 (15)	0.33582 (12)	0.0346 (7)	
C13	0.14243 (15)	0.04930 (15)	0.28740 (12)	0.0364 (7)	
C14	0.10969 (15)	0.12467 (15)	0.31610 (12)	0.0347 (7)	
C15	0.14608 (14)	0.15236 (14)	0.39315 (12)	0.0284 (6)	
C16	0.33102 (12)	0.32180 (13)	0.65973 (11)	0.0225 (5)	
C17	0.28642 (13)	0.40108 (14)	0.63137 (11)	0.0263 (6)	
C18	0.30953 (13)	0.48151 (14)	0.67087 (11)	0.0274 (6)	
C19	0.37744 (13)	0.48436 (13)	0.73951 (11)	0.0257 (6)	
C20	0.42279 (14)	0.40575 (14)	0.76760 (12)	0.0325 (7)	
C21	0.40007 (13)	0.32554 (14)	0.72791 (12)	0.0307 (6)	
C22	0.39888 (14)	0.57134 (14)	0.78239 (11)	0.0284 (6)	
C23	0.48580 (16)	0.64419 (16)	0.89787 (13)	0.0398 (8)	
C24A	0.42436 (19)	0.65330 (19)	0.94755 (16)	0.0411 (7)	0.870 (4)
C25A	0.4480 (2)	0.6382 (2)	1.02341 (17)	0.0411 (7)	0.870 (4)
C25B	0.4084 (13)	0.6533 (15)	1.0086 (12)	0.0411 (7)	0.130 (4)
C24B	0.4763 (12)	0.6242 (13)	0.9812 (9)	0.0411 (7)	0.130 (4)
O3	0.72389 (9)	0.11506 (11)	0.98995 (8)	0.0361 (5)	
O4	0.68567 (8)	0.10185 (10)	1.10449 (8)	0.0288 (4)	
N3	0.25961 (10)	0.13099 (11)	0.79692 (9)	0.0218 (5)	
N4	0.23280 (10)	0.12800 (10)	0.91440 (9)	0.0219 (5)	
C26	0.16917 (12)	0.12879 (12)	0.78295 (11)	0.0217 (5)	
C27	0.29565 (12)	0.12908 (12)	0.87613 (10)	0.0210 (5)	
C28	0.15348 (12)	0.12699 (12)	0.85661 (11)	0.0213 (5)	
C29	0.06818 (12)	0.12799 (13)	0.87721 (11)	0.0224 (5)	
C30	0.06319 (13)	0.09824 (14)	0.95100 (12)	0.0280 (6)	
C31	−0.01582 (14)	0.10342 (14)	0.97286 (13)	0.0323 (7)	
C32	−0.09136 (14)	0.13606 (14)	0.92102 (13)	0.0315 (7)	
C33	−0.08745 (14)	0.16429 (15)	0.84699 (13)	0.0338 (7)	
C34	−0.00862 (13)	0.16088 (14)	0.82550 (12)	0.0303 (7)	
C35	0.11146 (12)	0.12649 (13)	0.70215 (11)	0.0222 (5)	
C36	0.05572 (13)	0.05385 (14)	0.67653 (12)	0.0278 (6)	
C37	0.00288 (13)	0.05064 (15)	0.59948 (12)	0.0319 (6)	
C38	0.00694 (13)	0.11936 (15)	0.54765 (12)	0.0323 (7)	
C39	0.06174 (13)	0.19250 (15)	0.57282 (12)	0.0305 (6)	
C40	0.11339 (12)	0.19657 (14)	0.65014 (11)	0.0261 (6)	
C41	0.39055 (12)	0.12569 (12)	0.91330 (11)	0.0210 (5)	
C42	0.45320 (12)	0.13070 (13)	0.86928 (11)	0.0240 (6)	
C43	0.54253 (12)	0.12645 (13)	0.90681 (11)	0.0245 (6)	
C44	0.57150 (12)	0.11583 (13)	0.98811 (11)	0.0226 (6)	
C45	0.50929 (12)	0.10990 (13)	1.03207 (11)	0.0240 (6)	
C46	0.42025 (12)	0.11466 (13)	0.99490 (11)	0.0242 (6)	
C47	0.66770 (13)	0.11106 (13)	1.02562 (11)	0.0259 (6)	
C48	0.77829 (13)	0.10768 (15)	1.14775 (12)	0.0324 (7)	
C49	0.80374 (15)	0.20428 (16)	1.15957 (13)	0.0384 (7)	
C50	0.86523 (17)	0.2423 (2)	1.13349 (15)	0.0527 (9)	
H5	0.38720	0.00130	0.71410	0.0320*	
H1N	0.2592 (13)	0.2721 (15)	0.5047 (12)	0.0270*	
H8	0.18040	−0.19070	0.55520	0.0420*	
H6	0.38420	−0.15050	0.74640	0.0360*	
H7	0.28350	−0.24790	0.66520	0.0380*	
H12	0.23390	−0.04990	0.31640	0.0420*	
H13	0.11740	0.02980	0.23490	0.0440*	
H14	0.06230	0.15750	0.28310	0.0420*	
H15	0.12240	0.20340	0.41280	0.0340*	
H17	0.23970	0.39980	0.58450	0.0320*	
H18	0.27870	0.53500	0.65090	0.0330*	
H20	0.46970	0.40720	0.81440	0.0390*	
H21	0.43190	0.27240	0.74740	0.0370*	
H23A	0.54690	0.63970	0.93170	0.0480*	
H23B	0.48200	0.69820	0.86440	0.0480*	
H24A	0.36520	0.67080	0.92370	0.0490*	0.870 (4)
H25A	0.50690	0.62070	1.04830	0.0490*	0.870 (4)
H25B	0.40640	0.64480	1.05370	0.0490*	0.870 (4)
H9	0.17810	−0.03840	0.52560	0.0360*	
H11	0.29870	−0.00090	0.44410	0.0350*	
H24B	0.52090	0.58930	1.01550	0.0490*	0.130 (4)
H25C	0.36280	0.68840	0.97570	0.0490*	0.130 (4)
H25D	0.40630	0.63880	1.06060	0.0490*	0.130 (4)
H3N	0.2875 (14)	0.1334 (14)	0.7588 (12)	0.0260*	
H30	0.11420	0.07420	0.98680	0.0340*	
H31	−0.01780	0.08430	1.02390	0.0390*	
H32	−0.14520	0.13910	0.93600	0.0380*	
H33	−0.13920	0.18620	0.81070	0.0410*	
H34	−0.00690	0.18130	0.77480	0.0360*	
H36	0.05370	0.00610	0.71180	0.0330*	
H37	−0.03590	0.00130	0.58260	0.0380*	
H38	−0.02790	0.11650	0.49470	0.0390*	
H39	0.06400	0.23990	0.53720	0.0370*	
H40	0.15010	0.24730	0.66750	0.0310*	
H42	0.43440	0.13700	0.81360	0.0290*	
H43	0.58450	0.13080	0.87660	0.0290*	
H45	0.52830	0.10260	1.08760	0.0290*	
H46	0.37850	0.11040	1.02530	0.0290*	
H48A	0.81530	0.07740	1.11780	0.0390*	
H48B	0.78760	0.07740	1.19940	0.0390*	
H49	0.77300	0.24050	1.18810	0.0460*	
H50A	0.89730	0.20800	1.10480	0.0630*	
H50B	0.87800	0.30420	1.14320	0.0630*	

### Atomic displacement parameters (Å^2^)

**Table:**

	*U*^11^	*U*^22^	*U*^33^	*U*^12^	*U*^13^	*U*^23^
O1	0.0718 (12)	0.0279 (9)	0.0318 (9)	0.0067 (8)	0.0000 (8)	−0.0006 (7)
O2	0.0383 (9)	0.0366 (9)	0.0356 (9)	−0.0007 (7)	0.0002 (7)	−0.0106 (7)
N1	0.0252 (8)	0.0227 (8)	0.0204 (8)	−0.0005 (7)	0.0069 (7)	0.0019 (7)
N2	0.0224 (8)	0.0248 (8)	0.0228 (8)	0.0017 (7)	0.0075 (7)	0.0001 (7)
C1	0.0235 (10)	0.0232 (10)	0.0234 (10)	0.0005 (8)	0.0086 (8)	0.0001 (8)
C2	0.0195 (9)	0.0273 (10)	0.0204 (9)	0.0014 (8)	0.0063 (7)	0.0022 (8)
C3	0.0200 (9)	0.0254 (10)	0.0229 (9)	0.0009 (8)	0.0074 (7)	0.0009 (8)
C4	0.0245 (10)	0.0252 (10)	0.0241 (10)	0.0025 (8)	0.0111 (8)	0.0022 (8)
C5	0.0272 (10)	0.0304 (11)	0.0228 (10)	0.0010 (8)	0.0060 (8)	0.0004 (8)
C6	0.0308 (11)	0.0342 (12)	0.0247 (10)	0.0060 (9)	0.0073 (8)	0.0075 (9)
C7	0.0308 (11)	0.0262 (11)	0.0407 (12)	0.0008 (9)	0.0120 (9)	0.0074 (9)
C8	0.0272 (11)	0.0318 (12)	0.0418 (13)	−0.0059 (9)	0.0019 (9)	0.0052 (10)
C9	0.0239 (10)	0.0298 (11)	0.0333 (11)	−0.0010 (8)	0.0024 (8)	0.0060 (9)
C10	0.0282 (10)	0.0240 (10)	0.0231 (10)	−0.0041 (8)	0.0091 (8)	0.0010 (8)
C11	0.0285 (10)	0.0297 (11)	0.0303 (11)	−0.0002 (9)	0.0102 (9)	−0.0014 (9)
C12	0.0418 (13)	0.0328 (12)	0.0338 (12)	−0.0046 (10)	0.0181 (10)	−0.0076 (9)
C13	0.0480 (14)	0.0401 (13)	0.0214 (10)	−0.0127 (11)	0.0094 (10)	−0.0050 (9)
C14	0.0426 (13)	0.0333 (12)	0.0246 (11)	−0.0031 (10)	0.0022 (9)	0.0033 (9)
C15	0.0356 (11)	0.0230 (10)	0.0266 (10)	−0.0002 (8)	0.0080 (9)	0.0001 (8)
C16	0.0229 (9)	0.0237 (10)	0.0228 (9)	−0.0016 (8)	0.0095 (8)	−0.0010 (8)
C17	0.0274 (10)	0.0320 (11)	0.0185 (9)	0.0031 (8)	0.0045 (8)	0.0011 (8)
C18	0.0331 (11)	0.0258 (10)	0.0228 (10)	0.0064 (9)	0.0062 (8)	0.0020 (8)
C19	0.0292 (10)	0.0271 (11)	0.0226 (10)	−0.0003 (8)	0.0102 (8)	0.0008 (8)
C20	0.0315 (11)	0.0323 (12)	0.0280 (11)	0.0000 (9)	−0.0025 (9)	0.0008 (9)
C21	0.0304 (11)	0.0260 (11)	0.0314 (11)	0.0035 (9)	0.0002 (9)	0.0031 (9)
C22	0.0361 (11)	0.0285 (11)	0.0222 (10)	−0.0024 (9)	0.0103 (9)	0.0003 (8)
C23	0.0437 (14)	0.0401 (13)	0.0356 (12)	−0.0095 (11)	0.0104 (10)	−0.0096 (10)
C24A	0.0403 (13)	0.0466 (12)	0.0377 (10)	−0.0018 (10)	0.0126 (9)	−0.0011 (9)
C25A	0.0403 (13)	0.0466 (12)	0.0377 (10)	−0.0018 (10)	0.0126 (9)	−0.0011 (9)
C25B	0.0403 (13)	0.0466 (12)	0.0377 (10)	−0.0018 (10)	0.0126 (9)	−0.0011 (9)
C24B	0.0403 (13)	0.0466 (12)	0.0377 (10)	−0.0018 (10)	0.0126 (9)	−0.0011 (9)
O3	0.0212 (7)	0.0556 (10)	0.0318 (8)	−0.0028 (7)	0.0075 (6)	−0.0056 (7)
O4	0.0203 (7)	0.0389 (8)	0.0247 (7)	−0.0016 (6)	0.0014 (5)	0.0012 (6)
N3	0.0196 (8)	0.0270 (9)	0.0190 (8)	−0.0001 (7)	0.0052 (6)	−0.0012 (6)
N4	0.0203 (8)	0.0246 (8)	0.0209 (8)	0.0003 (6)	0.0058 (6)	−0.0007 (6)
C26	0.0191 (9)	0.0212 (9)	0.0243 (10)	−0.0011 (7)	0.0049 (7)	−0.0004 (7)
C27	0.0213 (9)	0.0217 (9)	0.0196 (9)	0.0003 (7)	0.0046 (7)	−0.0011 (7)
C28	0.0213 (9)	0.0194 (9)	0.0232 (9)	0.0003 (7)	0.0056 (7)	−0.0010 (7)
C29	0.0201 (9)	0.0217 (9)	0.0265 (10)	−0.0024 (7)	0.0080 (8)	−0.0049 (8)
C30	0.0258 (10)	0.0310 (11)	0.0282 (10)	−0.0021 (8)	0.0087 (8)	0.0015 (9)
C31	0.0351 (12)	0.0338 (12)	0.0332 (11)	−0.0047 (9)	0.0182 (9)	0.0000 (9)
C32	0.0255 (10)	0.0296 (11)	0.0446 (13)	−0.0025 (9)	0.0182 (9)	−0.0063 (9)
C33	0.0235 (10)	0.0374 (12)	0.0406 (12)	0.0048 (9)	0.0085 (9)	−0.0018 (10)
C34	0.0282 (11)	0.0348 (12)	0.0293 (11)	0.0026 (9)	0.0100 (9)	−0.0004 (9)
C35	0.0167 (9)	0.0295 (10)	0.0204 (9)	0.0026 (8)	0.0049 (7)	−0.0021 (8)
C36	0.0263 (10)	0.0299 (11)	0.0265 (10)	0.0010 (8)	0.0058 (8)	−0.0005 (8)
C37	0.0245 (10)	0.0354 (12)	0.0323 (11)	−0.0020 (9)	0.0011 (9)	−0.0091 (9)
C38	0.0234 (10)	0.0485 (14)	0.0225 (10)	0.0065 (9)	0.0018 (8)	−0.0050 (9)
C39	0.0239 (10)	0.0435 (13)	0.0248 (10)	0.0074 (9)	0.0076 (8)	0.0076 (9)
C40	0.0197 (9)	0.0320 (11)	0.0275 (10)	0.0012 (8)	0.0077 (8)	0.0017 (8)
C41	0.0197 (9)	0.0213 (9)	0.0216 (9)	0.0003 (7)	0.0045 (7)	−0.0008 (7)
C42	0.0241 (10)	0.0297 (11)	0.0180 (9)	−0.0015 (8)	0.0054 (8)	0.0008 (8)
C43	0.0222 (9)	0.0286 (10)	0.0243 (10)	−0.0030 (8)	0.0090 (8)	−0.0015 (8)
C44	0.0199 (9)	0.0236 (10)	0.0238 (10)	−0.0014 (8)	0.0046 (7)	−0.0024 (8)
C45	0.0229 (10)	0.0287 (10)	0.0197 (9)	−0.0009 (8)	0.0046 (7)	−0.0026 (8)
C46	0.0235 (10)	0.0283 (10)	0.0228 (10)	0.0006 (8)	0.0098 (8)	−0.0004 (8)
C47	0.0243 (10)	0.0270 (10)	0.0267 (10)	−0.0024 (8)	0.0071 (8)	−0.0039 (8)
C48	0.0233 (10)	0.0404 (13)	0.0294 (11)	−0.0006 (9)	−0.0002 (8)	0.0019 (9)
C49	0.0341 (12)	0.0478 (14)	0.0296 (12)	−0.0069 (10)	0.0017 (9)	−0.0036 (10)
C50	0.0543 (16)	0.0612 (18)	0.0420 (14)	−0.0221 (14)	0.0115 (12)	−0.0068 (13)

### Geometric parameters (Å, º)

**Table:**

O1—C22	1.205 (3)	C20—H20	0.9500
O2—C22	1.338 (3)	C21—H21	0.9500
O2—C23	1.470 (3)	C23—H23A	0.9900
O3—C47	1.211 (3)	C23—H23B	0.9900
O4—C48	1.457 (3)	C24A—H24A	0.9500
O4—C47	1.348 (2)	C24B—H24B	0.9500
N1—C1	1.382 (3)	C25A—H25A	0.9500
N1—C2	1.361 (2)	C25A—H25B	0.9500
N2—C3	1.384 (3)	C25B—H25C	0.9500
N2—C2	1.331 (3)	C25B—H25D	0.9500
N1—H1N	0.90 (2)	C26—C35	1.473 (3)
N3—C27	1.360 (2)	C26—C28	1.381 (3)
N3—C26	1.377 (3)	C27—C41	1.464 (3)
N4—C27	1.332 (3)	C28—C29	1.476 (3)
N4—C28	1.387 (3)	C29—C34	1.396 (3)
N3—H3N	0.89 (2)	C29—C30	1.393 (3)
C1—C10	1.475 (3)	C30—C31	1.394 (3)
C1—C3	1.389 (3)	C31—C32	1.382 (3)
C2—C16	1.467 (3)	C32—C33	1.385 (3)
C3—C4	1.475 (3)	C33—C34	1.387 (3)
C4—C9	1.398 (3)	C35—C40	1.393 (3)
C4—C5	1.398 (3)	C35—C36	1.390 (3)
C5—C6	1.391 (3)	C36—C37	1.393 (3)
C6—C7	1.385 (3)	C37—C38	1.383 (3)
C7—C8	1.384 (3)	C38—C39	1.387 (3)
C8—C9	1.384 (3)	C39—C40	1.391 (3)
C10—C15	1.393 (3)	C41—C42	1.404 (3)
C10—C11	1.401 (3)	C41—C46	1.398 (3)
C11—C12	1.385 (3)	C42—C43	1.387 (3)
C12—C13	1.385 (3)	C43—C44	1.392 (3)
C13—C14	1.384 (3)	C44—C45	1.398 (3)
C14—C15	1.389 (3)	C44—C47	1.484 (3)
C16—C17	1.396 (3)	C45—C46	1.382 (3)
C16—C21	1.392 (3)	C48—C49	1.493 (3)
C17—C18	1.384 (3)	C49—C50	1.302 (4)
C18—C19	1.387 (3)	C30—H30	0.9500
C19—C22	1.492 (3)	C31—H31	0.9500
C19—C20	1.392 (3)	C32—H32	0.9500
C20—C21	1.382 (3)	C33—H33	0.9500
C23—C24B	1.541 (16)	C34—H34	0.9500
C23—C24A	1.468 (4)	C36—H36	0.9500
C24A—C25A	1.308 (4)	C37—H37	0.9500
C24B—C25B	1.35 (3)	C38—H38	0.9500
C5—H5	0.9500	C39—H39	0.9500
C6—H6	0.9500	C40—H40	0.9500
C7—H7	0.9500	C42—H42	0.9500
C8—H8	0.9500	C43—H43	0.9500
C9—H9	0.9500	C45—H45	0.9500
C11—H11	0.9500	C46—H46	0.9500
C12—H12	0.9500	C48—H48A	0.9900
C13—H13	0.9500	C48—H48B	0.9900
C14—H14	0.9500	C49—H49	0.9500
C15—H15	0.9500	C50—H50A	0.9500
C17—H17	0.9500	C50—H50B	0.9500
C18—H18	0.9500		
			
C22—O2—C23	116.69 (17)	C23—C24A—H24A	119.00
C47—O4—C48	116.30 (15)	C25B—C24B—H24B	118.00
C1—N1—C2	108.00 (16)	C23—C24B—H24B	118.00
C2—N2—C3	106.02 (15)	C24A—C25A—H25A	120.00
C1—N1—H1N	125.1 (14)	C24A—C25A—H25B	120.00
C2—N1—H1N	126.3 (14)	H25A—C25A—H25B	120.00
C26—N3—C27	108.42 (16)	H25C—C25B—H25D	120.00
C27—N4—C28	105.78 (15)	C24B—C25B—H25D	120.00
C27—N3—H3N	128.0 (14)	C24B—C25B—H25C	120.00
C26—N3—H3N	123.6 (14)	N3—C26—C35	121.24 (17)
N1—C1—C3	105.27 (16)	N3—C26—C28	105.17 (16)
C3—C1—C10	133.88 (18)	C28—C26—C35	133.57 (18)
N1—C1—C10	120.86 (17)	N3—C27—C41	124.12 (17)
N1—C2—N2	111.01 (17)	N4—C27—C41	125.19 (16)
N1—C2—C16	124.12 (17)	N3—C27—N4	110.66 (16)
N2—C2—C16	124.86 (17)	C26—C28—C29	128.75 (18)
N2—C3—C1	109.68 (17)	N4—C28—C26	109.94 (17)
C1—C3—C4	130.31 (18)	N4—C28—C29	121.25 (16)
N2—C3—C4	119.95 (16)	C30—C29—C34	117.99 (18)
C5—C4—C9	118.17 (18)	C28—C29—C34	121.91 (17)
C3—C4—C9	121.47 (17)	C28—C29—C30	120.07 (17)
C3—C4—C5	120.35 (17)	C29—C30—C31	120.69 (19)
C4—C5—C6	120.62 (19)	C30—C31—C32	120.7 (2)
C5—C6—C7	120.45 (19)	C31—C32—C33	119.1 (2)
C6—C7—C8	119.3 (2)	C32—C33—C34	120.5 (2)
C7—C8—C9	120.7 (2)	C29—C34—C33	121.08 (19)
C4—C9—C8	120.76 (19)	C26—C35—C36	120.49 (17)
C1—C10—C15	120.56 (18)	C36—C35—C40	119.32 (18)
C1—C10—C11	121.07 (18)	C26—C35—C40	120.18 (17)
C11—C10—C15	118.37 (18)	C35—C36—C37	120.44 (19)
C10—C11—C12	120.19 (19)	C36—C37—C38	119.9 (2)
C11—C12—C13	120.9 (2)	C37—C38—C39	120.14 (19)
C12—C13—C14	119.50 (19)	C38—C39—C40	120.1 (2)
C13—C14—C15	120.0 (2)	C35—C40—C39	120.15 (19)
C10—C15—C14	121.1 (2)	C42—C41—C46	118.59 (18)
C2—C16—C21	119.73 (18)	C27—C41—C46	119.36 (17)
C17—C16—C21	118.62 (18)	C27—C41—C42	122.03 (17)
C2—C16—C17	121.65 (17)	C41—C42—C43	120.17 (17)
C16—C17—C18	120.71 (18)	C42—C43—C44	120.84 (18)
C17—C18—C19	120.29 (19)	C45—C44—C47	121.90 (17)
C20—C19—C22	121.56 (18)	C43—C44—C45	119.17 (18)
C18—C19—C20	119.28 (18)	C43—C44—C47	118.93 (17)
C18—C19—C22	119.15 (18)	C44—C45—C46	120.13 (17)
C19—C20—C21	120.44 (19)	C41—C46—C45	121.10 (18)
C16—C21—C20	120.65 (19)	O3—C47—C44	124.24 (17)
O1—C22—C19	124.01 (19)	O3—C47—O4	123.59 (18)
O1—C22—O2	123.33 (19)	O4—C47—C44	112.17 (17)
O2—C22—C19	112.65 (17)	O4—C48—C49	108.94 (18)
O2—C23—C24B	110.7 (7)	C48—C49—C50	124.5 (2)
O2—C23—C24A	110.9 (2)	C29—C30—H30	120.00
C23—C24A—C25A	122.4 (3)	C31—C30—H30	120.00
C23—C24B—C25B	124.0 (15)	C30—C31—H31	120.00
C6—C5—H5	120.00	C32—C31—H31	120.00
C4—C5—H5	120.00	C31—C32—H32	120.00
C5—C6—H6	120.00	C33—C32—H32	121.00
C7—C6—H6	120.00	C32—C33—H33	120.00
C8—C7—H7	120.00	C34—C33—H33	120.00
C6—C7—H7	120.00	C29—C34—H34	120.00
C7—C8—H8	120.00	C33—C34—H34	119.00
C9—C8—H8	120.00	C35—C36—H36	120.00
C4—C9—H9	120.00	C37—C36—H36	120.00
C8—C9—H9	120.00	C36—C37—H37	120.00
C12—C11—H11	120.00	C38—C37—H37	120.00
C10—C11—H11	120.00	C37—C38—H38	120.00
C13—C12—H12	120.00	C39—C38—H38	120.00
C11—C12—H12	120.00	C38—C39—H39	120.00
C14—C13—H13	120.00	C40—C39—H39	120.00
C12—C13—H13	120.00	C35—C40—H40	120.00
C15—C14—H14	120.00	C39—C40—H40	120.00
C13—C14—H14	120.00	C41—C42—H42	120.00
C14—C15—H15	119.00	C43—C42—H42	120.00
C10—C15—H15	119.00	C42—C43—H43	120.00
C18—C17—H17	120.00	C44—C43—H43	120.00
C16—C17—H17	120.00	C44—C45—H45	120.00
C17—C18—H18	120.00	C46—C45—H45	120.00
C19—C18—H18	120.00	C41—C46—H46	119.00
C19—C20—H20	120.00	C45—C46—H46	119.00
C21—C20—H20	120.00	O4—C48—H48A	110.00
C16—C21—H21	120.00	O4—C48—H48B	110.00
C20—C21—H21	120.00	C49—C48—H48A	110.00
C24B—C23—H23A	75.00	C49—C48—H48B	110.00
C24B—C23—H23B	136.00	H48A—C48—H48B	108.00
O2—C23—H23B	109.00	C48—C49—H49	118.00
O2—C23—H23A	109.00	C50—C49—H49	118.00
H23A—C23—H23B	108.00	C49—C50—H50A	120.00
C24A—C23—H23A	109.00	C49—C50—H50B	120.00
C24A—C23—H23B	109.00	H50A—C50—H50B	120.00
C25A—C24A—H24A	119.00		
			
C22—O2—C23—C24A	80.2 (2)	C16—C17—C18—C19	−0.1 (3)
C23—O2—C22—O1	4.2 (3)	C17—C18—C19—C22	−177.89 (19)
C23—O2—C22—C19	−174.53 (18)	C17—C18—C19—C20	0.9 (3)
C48—O4—C47—C44	−172.67 (16)	C20—C19—C22—O2	0.8 (3)
C47—O4—C48—C49	81.7 (2)	C22—C19—C20—C21	178.2 (2)
C48—O4—C47—O3	7.2 (3)	C18—C19—C22—O2	179.55 (18)
C2—N1—C1—C3	1.7 (2)	C20—C19—C22—O1	−177.9 (2)
C1—N1—C2—N2	−1.1 (2)	C18—C19—C22—O1	0.8 (3)
C2—N1—C1—C10	−177.97 (18)	C18—C19—C20—C21	−0.5 (3)
C1—N1—C2—C16	−179.83 (18)	C19—C20—C21—C16	−0.6 (3)
C3—N2—C2—C16	178.73 (18)	O2—C23—C24A—C25A	107.8 (3)
C2—N2—C3—C4	−176.20 (17)	N3—C26—C35—C40	−60.9 (3)
C3—N2—C2—N1	0.0 (2)	N3—C26—C28—N4	0.0 (2)
C2—N2—C3—C1	1.1 (2)	N3—C26—C35—C36	118.0 (2)
C26—N3—C27—N4	−1.6 (2)	C28—C26—C35—C40	121.2 (2)
C27—N3—C26—C28	0.9 (2)	C35—C26—C28—C29	−4.5 (3)
C27—N3—C26—C35	−177.55 (17)	C28—C26—C35—C36	−60.0 (3)
C26—N3—C27—C41	176.59 (17)	C35—C26—C28—N4	178.17 (19)
C28—N4—C27—C41	−176.64 (17)	N3—C26—C28—C29	177.26 (18)
C27—N4—C28—C29	−178.40 (17)	N3—C27—C41—C42	4.7 (3)
C27—N4—C28—C26	−0.9 (2)	N3—C27—C41—C46	−173.48 (18)
C28—N4—C27—N3	1.5 (2)	N4—C27—C41—C42	−177.41 (18)
C3—C1—C10—C15	139.4 (2)	N4—C27—C41—C46	4.4 (3)
N1—C1—C3—N2	−1.7 (2)	N4—C28—C29—C30	−23.0 (3)
C3—C1—C10—C11	−40.8 (3)	N4—C28—C29—C34	154.91 (18)
C10—C1—C3—C4	−5.2 (4)	C26—C28—C29—C30	160.0 (2)
N1—C1—C10—C15	−41.0 (3)	C26—C28—C29—C34	−22.1 (3)
N1—C1—C10—C11	138.8 (2)	C30—C29—C34—C33	0.3 (3)
N1—C1—C3—C4	175.22 (19)	C34—C29—C30—C31	−1.6 (3)
C10—C1—C3—N2	177.9 (2)	C28—C29—C34—C33	−177.64 (19)
N2—C2—C16—C17	−152.4 (2)	C28—C29—C30—C31	176.44 (18)
N1—C2—C16—C21	−153.30 (19)	C29—C30—C31—C32	1.7 (3)
N1—C2—C16—C17	26.2 (3)	C30—C31—C32—C33	−0.5 (3)
N2—C2—C16—C21	28.1 (3)	C31—C32—C33—C34	−0.8 (3)
C1—C3—C4—C9	−24.5 (3)	C32—C33—C34—C29	0.9 (3)
N2—C3—C4—C5	−26.6 (3)	C36—C35—C40—C39	−1.7 (3)
C1—C3—C4—C5	156.7 (2)	C26—C35—C36—C37	−178.30 (19)
N2—C3—C4—C9	152.21 (19)	C40—C35—C36—C37	0.6 (3)
C5—C4—C9—C8	−3.0 (3)	C26—C35—C40—C39	177.17 (18)
C3—C4—C5—C6	−179.84 (19)	C35—C36—C37—C38	1.1 (3)
C3—C4—C9—C8	178.20 (19)	C36—C37—C38—C39	−1.7 (3)
C9—C4—C5—C6	1.3 (3)	C37—C38—C39—C40	0.6 (3)
C4—C5—C6—C7	1.2 (3)	C38—C39—C40—C35	1.2 (3)
C5—C6—C7—C8	−2.0 (3)	C27—C41—C46—C45	179.10 (18)
C6—C7—C8—C9	0.4 (3)	C42—C41—C46—C45	0.8 (3)
C7—C8—C9—C4	2.2 (3)	C27—C41—C42—C43	−179.44 (18)
C1—C10—C11—C12	179.2 (2)	C46—C41—C42—C43	−1.2 (3)
C15—C10—C11—C12	−1.0 (3)	C41—C42—C43—C44	1.0 (3)
C1—C10—C15—C14	179.3 (2)	C42—C43—C44—C45	−0.3 (3)
C11—C10—C15—C14	−0.5 (3)	C42—C43—C44—C47	179.62 (18)
C10—C11—C12—C13	1.8 (3)	C43—C44—C47—O4	179.25 (17)
C11—C12—C13—C14	−1.0 (3)	C45—C44—C47—O3	179.3 (2)
C12—C13—C14—C15	−0.5 (3)	C45—C44—C47—O4	−0.8 (3)
C13—C14—C15—C10	1.3 (3)	C43—C44—C47—O3	−0.7 (3)
C2—C16—C17—C18	179.52 (19)	C43—C44—C45—C46	−0.1 (3)
C17—C16—C21—C20	1.3 (3)	C47—C44—C45—C46	180.00 (19)
C2—C16—C21—C20	−179.13 (19)	C44—C45—C46—C41	−0.2 (3)
C21—C16—C17—C18	−0.9 (3)	O4—C48—C49—C50	−120.0 (2)

### Hydrogen-bond geometry (Å, º)

Cg1, Cg2 and Cg3 are the centroids of the N1/N2/C1–C3 1*H*-imidazole ring and the C4–C9 and C10–C15 phenyl rings, respectively.

**Table:**

*D*—H···*A*	*D*—H	H···*A*	*D*···*A*	*D*—H···*A*
N1—H1*N*···N4^i^	0.90 (2)	2.14 (2)	3.037 (2)	176 (2)
N3—H3*N*···N2	0.89 (2)	2.06 (2)	2.925 (2)	164 (2)
C7—H7···O1^ii^	0.95	2.45	3.254 (3)	142
C20—H20···O2	0.95	2.41	2.736 (3)	100
C32—H32···O3^iii^	0.95	2.51	3.439 (3)	167
C46—H46···N4	0.95	2.60	2.921 (3)	100
C23—H23*A*···*Cg*1^iv^	0.99	2.74	3.524 (3)	136
C25*A*—H25*B*···*Cg*2^v^	0.95	2.79	3.687 (3)	158
C37—H37···*Cg*3^vi^	0.95	2.90	3.634 (3)	134
C25*B*—H25*D*···*Cg*2^v^	0.95	2.68	3.44 (2)	137

Symmetry codes: (i) *x*, −*y*+1/2, *z*−1/2; (ii) *x*, *y*−1, *z*; (iii) *x*−1, *y*, *z*; (iv) −*x*+1, *y*+1/2, −*z*+3/2; (v) *x*, −*y*+1/2, *z*+1/2; (vi) −*x*, −*y*, −*z*+1.
